# Enhancing Photoluminescence Quenching in Donor–Acceptor PCE11:PPCBMB Films through the Optimization of Film Microstructure

**DOI:** 10.3390/nano9121757

**Published:** 2019-12-10

**Authors:** Otto Todor-Boer, Ioan Petrovai, Raluca Tarcan, Adriana Vulpoi, Leontin David, Simion Astilean, Ioan Botiz

**Affiliations:** 1 Interdisciplinary Research Institute in Bio-Nano-Sciences, Babes-Bolyai University, Treboniu Laurian 42, 400271 Cluj-Napoca, Romania; otto.todor@icia.ro (O.T.-B.); ioan.petrovai@ubbonline.ubbcluj.ro (I.P.); tara13456@studmail.ubbcluj.ro (R.T.); adriana.vulpoi@phys.ubbcluj.ro (A.V.); simion.astilean@phys.ubbcluj.ro (S.A.); 2 Faculty of Physics, Babes-Bolyai University, M. Kogalniceanu Str. 1, 400084 Cluj-Napoca, Romania; leontin.david@phys.ubbcluj.ro; 3 INCDO-INOE 2000, Research Institute for Analytical Instrumentation, Donath Street 67, 400293 Cluj-Napoca, Romania

**Keywords:** conjugated polymers, polyfullerenes, processing by convective self-assembly, thin films and microstructure, photoluminescence quenching

## Abstract

We show that a precise control of deposition speed during the fabrication of polyfullerenes and donor polymer films by convective self-assembly leads to an optimized film microstructure comprised of interconnected crystalline polymer domains comparable to molecular dimensions intercalated with similar polyfullerene domains. Moreover, in blended films, we have found a correlation between deposition speed, the resulting microstructure, and photoluminescence quenching. The latter appeared more intense for lower deposition speeds due to a more favorable structuring at the nanoscale of the two donor and acceptor systems in the resulting blend films.

## 1. Introduction

Nowadays, alternative renewable energy sources are becoming essential in our society, and organic photovoltaics (OPVs) could become in the near future a viable technological solution for continuously increasing societal energy needs. OPVs are based on semiconducting conjugated materials, and at the moment, they can convert solar energy into electricity with a power conversion efficiency of about 16.5% [1]. Researchers have shown in this last decade that in order to make efficient OPVs, one has to manipulate a series of internal fundamental phenomena that occur in the active layer such as exciton diffusion and separation [2,3], charge transport and carrier migration [4,5], or exciton and charge recombination [6] directly by controlling the microstructure of the active layer from nano- to micro- and up to the macroscale [4,6,7,8,9,10]. Thus, a fine control over the microstructure in the active layer is expected to tune the resulting optoelectronic properties, including the emission properties. For instance, because photoluminescence (PL) quenching is a measure of excitons separation at the donor–acceptor interfaces [11], it is important to maximize PL quenching between the donor and the acceptor materials when fabricating OPVs.

In the literature, researchers have described many efficient techniques that can be used to control more or less molecular conformations and microstructure in the active layer [12,13,14,15,16,17,18,19,20] and thus, to efficiently tune the emission/quenching properties [21,22]. These techniques also include convective self-assembly (CSA), which is a blade coating type technique [23] that can permit the specific control of the backbone/chain conformation by favoring more planarized polymer structures [24]. CSA is a specific methodology that is used to deposit colloidal solutions onto solid substrates. With CSA, by precisely controlling the deposition temperature and the specific tilt angle of the deposition ‘blade’ of the used coater, the forces acting at the triple air–substrate–solution interface can be manipulated while the solution is cast onto a carrier moving with controlled speed. As a consequence, CSA can allow us to control solvent evaporation rate and thus can be adapted to fabricate uniform, structured films using both oligomeric and macromolecular species.

**Figure 1:**
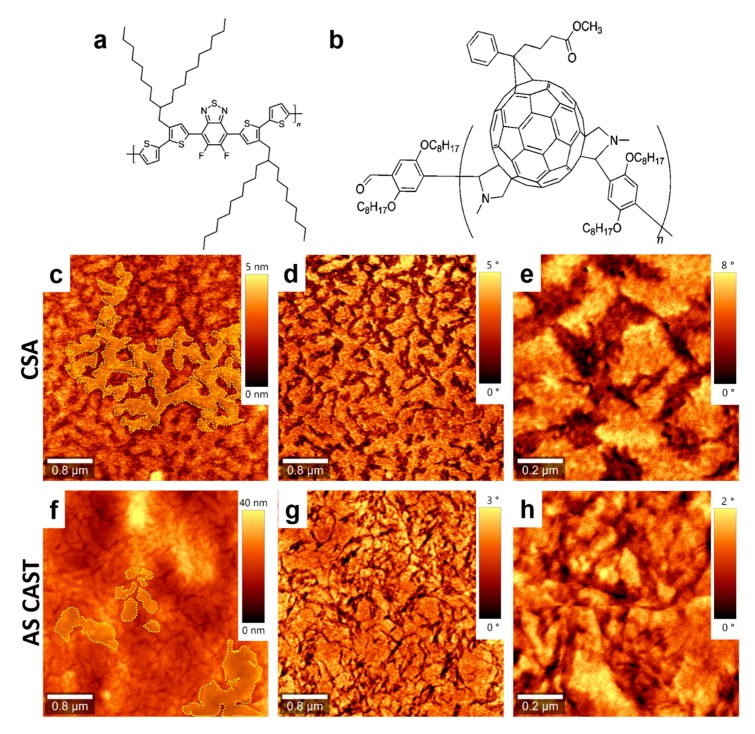
(**a**,**b**) Chemical structure of poly[(5,6-difluoro-2,1,3-benzothiadiazol-4,7-diyl)-alt-(3,3′-di(2-octyldodecyl)2,2′;5′,2′; 5′,2′-quaterthiophen-5,5′-diyl)] (PCE11) (**a**) and poly{[bispyrrolidino (phenyl-C61-butyric acid methyl ester)]-alt-[2,5-bis(octyloxy)benzene]} (PPCBMB) (**b**) systems. Topography (**c**) and phase (**d**) atomic force microscopy (AFM) images of a PCE11:PPCBMB thin film prepared using convective self-assembly (CSA) at a casting speed of 10 µm/s. (**e**) Zoom-in of the image shown in (**d**). Topography (**f**) and phase (**g**) AFM images of a PCE11:PPCBMB as spin cast film. (**h**) Zoom-in of the image shown in (**g**). Dotted lines are for eye guiding only and are indicating various PCE11 structures.

Here, we employ CSA technique at various deposition speeds to produce donor–acceptor thin films of poly[(5,6-difluoro-2,1,3-benzothiadiazol-4,7-diyl)-alt-(3,3′-di(2-octyldodecyl)2,2′;5′,2′; 5′,2′-quaterthiophen-5,5′-diyl)] (PCE11; Figure 1a) and poly{[bispyrrolidino (phenyl-C61-butyric acid methyl ester)]-alt-[2,5-bis(octyloxy)benzene]} (PPCBMB; Figure 1b) that exhibit much higher PL quenching than their PCE11:PPCBMB analogues made by the spin-casting technique. As revealed by atomic force microscopy (AFM), this higher PL quenching is related to the changes in film microstructure induced upon film deposition using CSA. Our results are of importance in the OPVs field, as PCE11 and fullerenes are promising materials for organic solar cell fabrication [25,26,27]. 

## 2. Materials and Methods 

PCE11 of a weight-average molecular weight $M_{w}\;=\;112.707\;\mathrm{kg}/\mathrm{mol}$, number-average molecular weight $M_{n}\;=\;55.674\;\mathrm{kg}/\mathrm{mol}$, and polydispersity index PDI = 2.02 was purchased from Ossila Ltd. (Sheffield, UK) PPCBMB of a weight-average molecular weight $M_{w}\;\approx \;73.8\;\mathrm{kg}/\mathrm{mol}$, number-average molecular weight $M_{n}\;\approx \;24.6\;\mathrm{kg}/\mathrm{mol}$ and poly dispersity index PDI = 3 was obtained from sterically controlled azomethine ylide cycloaddition polymerization of the phenyl-C61-butyric acid methyl ester (PCBM) as described elsewhere [28].

Thin films of PCE11 and PPCBMB were made by spin casting at 2000 rpm from 6 g/L chlorobenzene solution (with the resulting film thickness of about 80 ± 8 nm) as well as by CSA at deposition speeds of 1000 µm/s, 500 µm/s, 100 µm/s, 50 µm/s, 25 µm/s, and 10 µm/s respectively (with the resulting film thickness varying between 30 ± 5 nm and 140 ± 15 nm, respectively). A 50/50 weight percentage ratio was used to obtain solutions of PCE11:PPCBMB blend. Films of PCE11:PPCBMB were also made employing the above described procedure. For all films, regular UV–ozone cleaned microscopy cover glass was used as substrate.

The CSA coater was comprised of a motorized translation stage using a linear actuator from Zaber Technologies and moving with speeds ranging between ~4.7 μm/s and 8 mm/s. A temperature controller was placed on top of the translation stage, and the temperature of the substrate was regulated between 17 °C and 24 °C using a water-controlled system (Accel 250 LC from Thermo Scientific). A cover glass that acted as a blade was fixed in the near vicinity of the substrate at the desired angle; the polymer solution was placed on the substrate, underneath and near the edge of the blade. This configuration allowed us to precisely control both the deposition speed and the temperature of the substrate.

For the acquisition of AFM images, an Alpha 300A microscope from Witec was used in tapping mode. PL spectra were collected using an FP-6500 Spectrofluorometer from Jasco (excitation wavelength range of 220–750 nm). All PL spectra were recorded using an excitation wavelength of 640 nm. 

## 3. Results and Discussion

Most of the work performed on OPVs has focused on film microstructures resulted by blending conjugated donor polymers with various fullerenes. Obtained microstructures most often were characterized by the existence of randomly alternating donor and acceptor domains, each greatly varying in size. Aiming to gain control over molecular packing at the nanoscale, we have decided in this work to replace fullerenes with polyfullerenes and to blend them with a crystalline PCE11 polymer system. This way, we expected to stimulate the phase separation process between polymer and polyfullerenes and thus, under specific CSA casting conditions, to control the size of resulting alternating donor–acceptor domains.

Indeed, the deposition of PCE11:PPCBMB blended films using the CSA technique led to a film microstructure that could be differentiated from that obtained in spin-cast analogue films (Figure 1). On the surface of the film prepared by CSA, brighter crystalline regions are alternating with wide darker regions (Figure 1c–e). We attribute these crystalline structures to the electron donor PCE11, which is a polymer system that is known for its high crystallinity and preferential face-on orientation of domains [25]. Note that generally, crystalline phases can be identified when using both AFM topographic (height mode) and viscoelastic (phase mode) images simultaneously [29,30,31]. Topography involves giving information about the surface profile/roughness while the phase is able to judge the structure of different material phases such as material softness/stiffness. As PCE11 is crystalline, i.e., stiffer material, it appears brighter in AFM phase images. Darker, most probably amorphous regions correspond to the electron acceptor PPCBMB. Moreover, these narrow crystalline domains displaying several tens of nanometers in lateral size are both randomly oriented and well-interconnected, forming complex crystalline networks (an example of such a network is delimited by dotted lines in Figure 1c) surrounded by PPCBMB regions. Instead, when analyzing the spin-cast film, we have noticed the presence of crystalline structures of various sizes ranging from very small (~0.05 ± 0.02 µm^2^) to much bigger structures (~0.55 ± 0.2 µm^2^). No matter their size, these structures were separated from each other and surrounded by much narrower PPCBMB regions (Figure 1f–h). Therefore, they appeared rather disconnected, as indicated by the dotted lines in Figure 1f.

**Figure 2:**
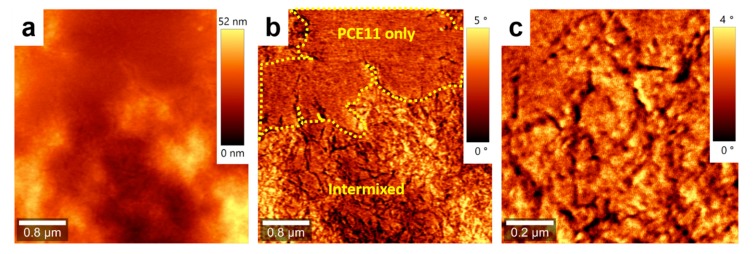
Topography (**a**) and phase (**b**) AFM images of a PCE11:PPCBMB thin film prepared by CSA at a deposition speed of 1000 µm/s. (**c**) Zoom-in of an intermixed region shown in (**b**).

Furthermore, the ratio between the area of crystalline PCE11 (~57% of the total surface) and the area of amorphous PPCBMB (~43% of the total surface) regions was about 1.32 for the film produced using CSA. In comparison, for the spin-cast film, this ratio was about 4 (with 80% of the surface covered by crystalline regions and 20% of the surface covered by amorphous regions). Although AFM measurements do not exclude the existence in both films of regions with intercalated PCE11 and PPCBMB molecules, it appears that the microstructure optimized using CSA was roughly comprised of interconnected, crystalline electron donating domains displaying a width comparable to molecular dimensions that were intercalated with similar electron-accepting domains. The above described microstructure could be much altered by increasing the CSA casting speed to, for example, 1000 µm/s. In these conditions, micrometer large crystalline regions of pure PCE11 were formed, and they were separated from intermixed regions containing small structures of both PCE11 and PPCBMB systems (Figure 2).

**Figure 3:**
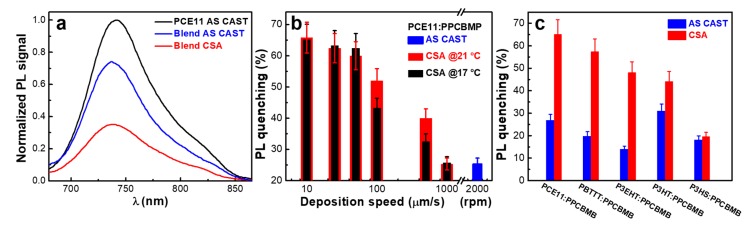
(**a**) Photoluminescence (PL) spectra of PCE11 and PCE11:PPCBMB films deposited by spin casting and CSA. (**b**) Amount of PL quenching in PCE11:PPCBMB films deposited by spin casting and CSA at 17 °C and 21 °C using various casting speeds. (**c**) Amount of PL quenching measured in various PPCBMB based blends that were deposited by spin casting and CSA at low casting speeds.

Since PL quenching is a measure of exciton separation at the donor–acceptor interfaces, we have further compared the emission properties of the two films presented in Figure 1. We have found that there was a 65% PL quenching in the film produced using CSA compared to only a 25% PL quenching measured for the spin-cast film (Figure 3a). There are two reasons that could explain these rather low PL quenching values measured for PCE11:PPCBMB films. One reason is related to the lower unoccupied molecular orbital (LUMO) of PCE11 (−3.69 eV [25]) and of PPCBMB (−3.34 eV [28]) systems that are not necessarily favoring charge transfer (nonetheless, charge transfer conditions could still be fulfilled, because LUMO levels could be slightly modified when blending the two systems [32]). The other reason is related to the fact that long chains of polyfullerenes are more difficult to intercalate in between PCE11 polymer chains to form PCE11:PPCBMB co-crystalline domains, which could favor excitons generation/separation due to close molecular packing [32].

Moreover, PL quenching was dependent on the CSA deposition speed (and less dependent on the temperature at which the substrate was kept during casting), i.e., on the type of the resulting microstructure (Figure 3b). Thus, we attribute this improvement of PL quenching from 25% to 65% to the optimization of the film microstructure through the realization of alternating donor and acceptor domains comparable to molecular dimensions and displaying a much larger interface area that favors stronger polymer–polyfullerene quencher interactions. Similar results were obtained for several other blends when replacing PCE11 with other conjugated polymer donor systems such as poly[2,5-bis(3-alkylthiophen-2-yl)thieno(3,2-b)thiophene] (PBTTT), poly(3-(2′-ethyl)hexyl-thiophene) (P3EHT), poly (3-hexylthiophene) (P3HT), or poly(3-hexyl)selenophene (P3HS) (Figure 3c), indicating an extended applicability of the CSA technique in the processing of PPCBMB-based blends.

**Figure 4:**
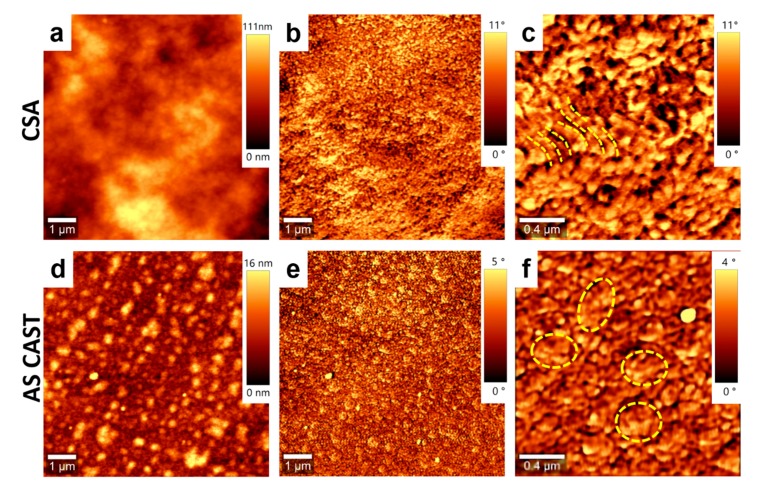
Topography (**a**) and phase (**b**) AFM images of a PCE11 thin film made by CSA at a low deposition speed of 10 µm/s. (**c**) Zoom-in of the image shown in (**b**). Topography (**d**) and phase (**e**) AFM images of a PCE11 as spin-cast film. (**f**) Zoom-in of the image shown in (**e**). Broken lines and shapes are for eye guiding only.

In order to further point out the efficiency of the CSA technique to induce structural changes in conjugated polymer/polyfullerene films, we have further investigated neat films of both PCE11 and PPCBMB. The results showed again that we can differentiate the microstructure of a PCE11 film that was deposited using CSA (Figure 4a–c) with respect to a PCE11 film that was simply spin cast (Figure 4d–f). The PCE11 film deposited using CSA at low casting speed displayed uniform granular structures that were often interconnected into rather elongated superstructures (indicated in Figure 4c by the broken lines) with a width of several tens of nanometers. Instead, the spin-cast film exhibited a microstructure that was comprised of many aggregated structures (indicated by the broken shapes in Figure 4f) randomly distributed on the surface along with much smaller granular structures. 

**Figure 5:**
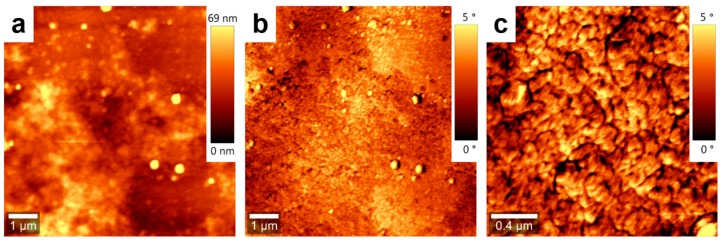
Topography (**a**) and phase (**b**) AFM images of a PCE11 thin film prepared by CSA at a deposition speed of 500 µm/s. (**c**) Zoom-in of the image shown in (**b**).

**Figure 6:**
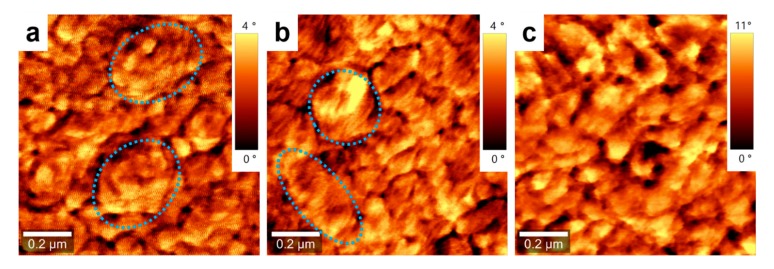
Comparison of phase AFM images of PCE11 thin films prepared by spin casting (**a**) and by CSA at a deposition speed of 500 µm/s (**b**) and of 10 µm/s (**c**). Dotted shapes are for eye guiding only.

The microstructure obtained at low casting CSA speeds could again be altered when increasing casting speed to, for instance, 500 µm/s (Figure 5). When we have looked into more details and compared high magnification AFM images of the three PCE11 films deposited by spin casting and by CSA at high and low deposition speeds respectively, we have noticed that the microstructure of PCE11 film deposited at high CSA speed was comprised of both bigger aggregates (indicated by dotted shapes) and smaller granular structures (Figure 6b). In this latter case, the obtained microstructure was similar to that observed for the as spin-cast PCE11 film (Figure 6a). In contrary, as already shown in Figure 4a–c, the higher magnification AFM image of PCE11 film prepared at low CSA speed emphasized even clearer the existence of a microstructure comprised of monomodal (interconnected) granular structures (Figure 6c).

**Figure 7:**
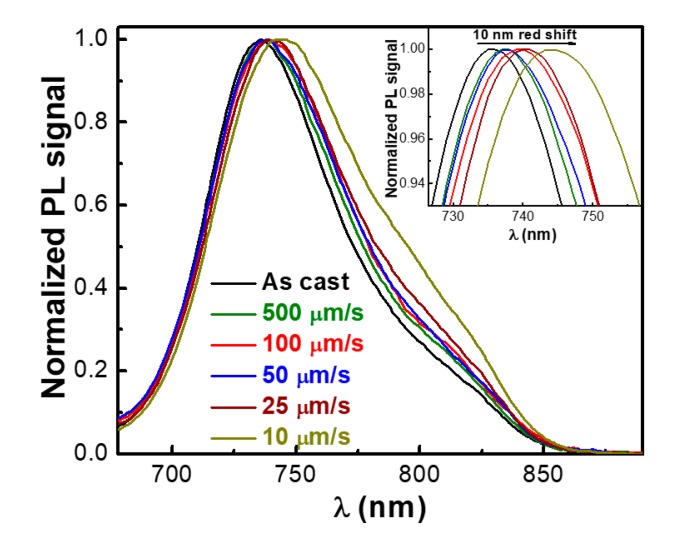
Normalized PL spectra recorded for PCE11 thin films deposited both by spin-casting and CSA techniques. The inset represents a zoom-in of the presented PL spectra.

The analysis of the PL spectra recorded for thin films of PCE11 deposited both by spin-casting and CSA techniques have further emphasized the differences in the film microstructures (Figure 7). We could notice the appearance of a red shift of ~10 nm in the main emission peak for films deposited very slowly using CSA in comparison to films spin cast or deposited via CSA but using higher casting speeds. According to the literature, these results are indicating at least that films deposited at higher speeds are comprised of polymer chains adopting less planarized (displaying more torsional disorder, i.e., shorter conjugation length) conformations of the backbone with excitons possibly experiencing a more disordered energy landscape than those cast at lower speeds [24].

**Figure 8:**
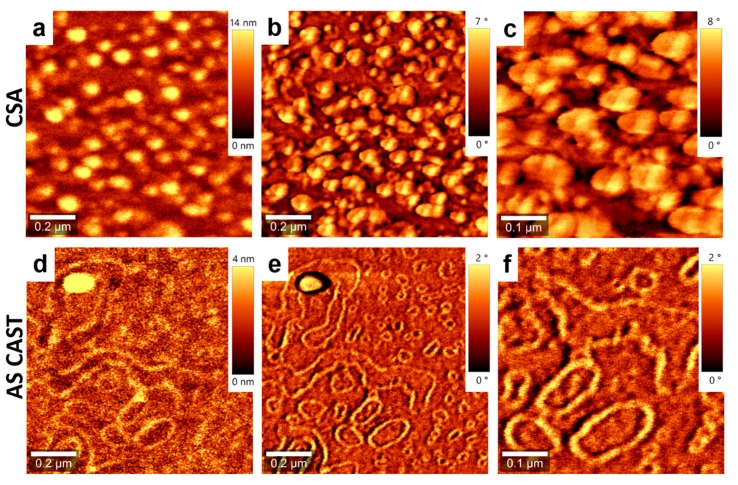
Topography (**a**) and phase (**b**) AFM images of a PPCBMB thin film made using CSA at a low deposition speed of 10 µm/s. (**c**) Zoom-in of the image shown in (**b**). Topography (**d**) and phase (**e**) AFM images of a PPCBMB as spin cast film. (**f**) Zoom-in of the image shown in (**e**).

**Figure 9:**
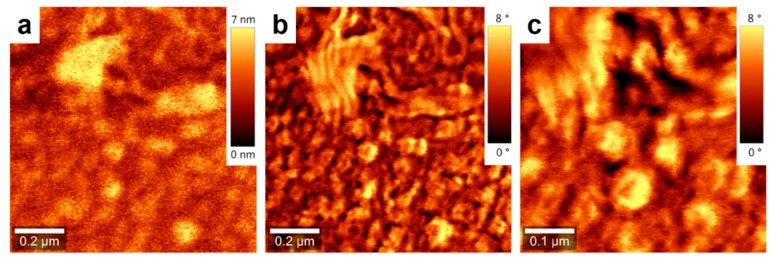
Topography (**a**) and phase (**b**) AFM images of a PPCBMB thin film prepared by CSA at a deposition speed of 1000 µm/s. (**c**) Zoom-in of the image shown in (**b**).

A further comparison of the microstructure between two films of PPCBMB obtained using CSA at low casting speed (Figure 8a–c) and spin casting (Figure 8d–f) also displayed structural differences. The microstructure of film prepared using CSA was comprised of disk-like structures of an average diameter of several tens of nanometers. Meanwhile, the microstructure of the spin-cast film was comprised of randomly distributed linear or circular fiber-like structures, with the average fiber width in the range of 20 ± 4 nm. When using CSA at higher casting speeds, a mixed microstructure comprised of both aggregated fiber-like and disk-like structures was obtained (Figure 9). Thus, similarly to PCE11, the PPCBMB system also exhibited differences in microstructure that were directly correlated to the casting techniques and conditions used for the preparation of thin films. Therefore, the above results indicate that the CSA technique is able to induce important microstructural changes in PCE11 and PPCBMB containing films, and thus that it can be efficiently used to control, for instance, PL quenching.

## 4. Conclusions

As revealed by AFM and PL spectroscopy, the quality of microstructure corresponding to thin films of PCE11 and PPCBMB depended on the casting conditions and technique. Only using CSA at low casting speed led to an optimized film microstructure that was comprised of crystalline domains of PCE11 alternated with PPCBMB amorphous regions and that exhibited strong PL quenching. These results indicate both that the microstructure of PCE11:PPCBMB films is highly sensitive to the conditions under which such films are prepared and that by carefully controlling the conditions of film preparation, we could finely tune the resulting optoelectronic properties as it is needed, for example, in OPV applications.
